# Combining Deep and Handcrafted Image Features for Presentation Attack Detection in Face Recognition Systems Using Visible-Light Camera Sensors

**DOI:** 10.3390/s18030699

**Published:** 2018-02-26

**Authors:** Dat Tien Nguyen, Tuyen Danh Pham, Na Rae Baek, Kang Ryoung Park

**Affiliations:** Division of Electronics and Electrical Engineering, Dongguk University, 30 Pildong-ro 1-gil, Jung-gu, Seoul 100-715, Korea; nguyentiendat@dongguk.edu (D.T.N.); phamdanhtuyen@gmail.com (T.D.P.); qorskfo1023@hanmail.net (N.R.B.)

**Keywords:** face recognition, presentation attack detection, multi-level local binary pattern, visible-light camera sensor, convolutional neural network

## Abstract

Although face recognition systems have wide application, they are vulnerable to presentation attack samples (fake samples). Therefore, a presentation attack detection (PAD) method is required to enhance the security level of face recognition systems. Most of the previously proposed PAD methods for face recognition systems have focused on using handcrafted image features, which are designed by expert knowledge of designers, such as Gabor filter, local binary pattern (LBP), local ternary pattern (LTP), and histogram of oriented gradients (HOG). As a result, the extracted features reflect limited aspects of the problem, yielding a detection accuracy that is low and varies with the characteristics of presentation attack face images. The deep learning method has been developed in the computer vision research community, which is proven to be suitable for automatically training a feature extractor that can be used to enhance the ability of handcrafted features. To overcome the limitations of previously proposed PAD methods, we propose a new PAD method that uses a combination of deep and handcrafted features extracted from the images by visible-light camera sensor. Our proposed method uses the convolutional neural network (CNN) method to extract deep image features and the multi-level local binary pattern (MLBP) method to extract skin detail features from face images to discriminate the real and presentation attack face images. By combining the two types of image features, we form a new type of image features, called hybrid features, which has stronger discrimination ability than single image features. Finally, we use the support vector machine (SVM) method to classify the image features into real or presentation attack class. Our experimental results indicate that our proposed method outperforms previous PAD methods by yielding the smallest error rates on the same image databases.

## 1. Introduction

Nowadays, biometric recognition systems are widely used in various application systems because they are hard to steal, have high recognition accuracy, and are convenient for users [1,2]. Such a recognition method is based on the difference in specific physical or behavioral characteristics among people. For example, previously, faces and/or fingerprints were used to distinguish people. Along with face and fingerprint, several biometric features, such as veins (blood vessels) [3,4], iris [2,5], palm-print [4,6,7], and ear [7,8], have been used in recognition applications. These previous studies have shown that biometric systems are more suitable in terms of enhancing recognition accuracy and user convenience as compared to traditional recognition methods, such as token-based methods (using keys and cards) or knowledge-based methods (using username and passwords). However, with the development of technology, biometric recognition systems have become vulnerable to fake samples being presented during the image acquisition process [1,9,10].

Among biometric recognition methods, face recognition is the most common and widely used method in applications such as computer/smartphone login, identification cards, and border and passport control [1,11,12]. This biometric feature uses the facial appearance as the key to recognize (distinguish) an individual among people. Although it has several drawbacks, such as variations in illumination and head pose, it is still used in combination with other biometric features (such as fingerprints, finger-veins, and palm-veins) to ensure the accuracy of recognition systems. In the face recognition process, users are required to present their faces in front of capturing devices (visible-light or thermal cameras) so that their face images can be captured. Subsequently, the face localization and feature extraction steps are performed to extract image features from the input face image. Finally, a matching step is performed to recognize (or identify) the user in the input image. Because of its operation procedure, a face recognition system can be attacked using printed photographs, masks, or video displays [13,14,15,16,17,18,19,20,21,22,23], thus reducing the security level of this system.

To solve this problem, the presentation attack detection (PAD) methods have been researched for face recognition system. Previous studies are classified into two categories of non-training-based and training-based feature extraction methods. As the former category, Tan et al. [14] used a sparse low-rank bilinear discriminative model on the image features extracted by a difference of Gaussian (DoG) and/or logarithmic total variation (LTV) methods to discriminate the real and presentation attack images. Using the NUAA database [14], they showed that the real and presentation attack images can be discriminated using their proposed method. With the NUAA database, Maatta et al. [15] used three feature extraction methods, Gabor filter, local phase quantization (LPQ), and local binary pattern (LBP), to extract the image features and classify the real and presentation attack images using support vector machines (SVMs). According to their results, the classification error was significantly reduced compared to those in research by Tan et al. [14]. Similarly, Benlamoudi et al. [20] used the LBP method for addressing the PAD problem for a face recognition system. However, they used the active shape model (ASM) method to align the face and the Fisher score to reduce the dimensionality of the extracted features. Parveen et al. [21] proposed a method that uses a dynamic local ternary pattern (DLTP) for detecting presentation attack face images; they used the DLTP method to extract image features and the SVM method for classification. However, the detection accuracy when using the NUAA database was slightly worse than those obtained by Maatta et al. [15] and Benlamoudi et al. [20], while the accuracy when using the CASIA database was better than that obtained by Benlamoudi et al. [20]. These results show that the handcrafted features are suitable for solving the PAD problem for a face recognition system. However, as shown in the above studies, the detection accuracy varies significantly among different databases, indicating that the handcrafted features do not completely solve the PAD problem.

The deep learning framework has shown very high classification accuracies compared to handcrafted features on many computer vision systems and has been successfully applied to various computer vision problems such as image classification [24,25,26,27], object detection [28,29], and face-based age estimation [30,31]. It has also been used for extracting image features for computer vision systems [32,33]. This method uses the image filtering technique to extract the image features and a neural network to classify the extracted image features into several desired classes. Using considerable training data, this method has proven to outperform conventional methods in computer vision systems. In addition, it has been successfully applied for detecting presentation attack images in finger-vein recognition systems [9].

Based on these consideration, training-based feature extraction methods have been studied as the second category of PAD methods. Menotti et al. [10] used such a method to detect presentation attack images in several biometric recognition systems such as iris, face, and fingerprint recognition systems. Their results indicated the sufficiency of the deep learning method for detecting presentation attack images in biometric recognition systems. However, their study also showed that this method did not outperform the handcrafted feature extraction method in all cases. In detail, the deep learning method outperformed the handcrafted feature extraction method in the case of the fingerprint recognition system, but not always in the cases of iris and face recognition systems.

In a study by Nanni et al. [34], the deep learning framework was applied for general image classification problem as an image feature extractor. In detail, they used several CNN models which were trained for several different problems to extract image features of the current problem. Based on the extracted image features, they used several SVM models to classify the input images into desired classes. Finally, the outputs of SVM models are combined with those based on handcrafted features using weighted sum rule to produce a final classification result. In another study [35], they additionally used several kinds of handcrafted image feature extraction methods such as LBP, local ternary pattern (LTP), LPQ to extract the image features besides the deep features for classification problem. As a result of this study, they proved that the handcrafted and deep image features can extract different information from input images. Based on this result, they showed that the combination of handcrafted and deep features is sufficient for enhancing the classification accuracy. However, the methods proposed in these studies use multiple CNN models and methods for handcrafted image feature extraction. This approach makes the classification system become very complex. In addition, the authors used only score level fusion with fixed weight values for combining the results of deep and handcrafted features. This could be a limitation of these methods because the weights should be selected according to the characteristics of images in each application. And, they did not apply their methods to the PAD problem for face recognition.

To overcome the above limitations of previous research on the PAD problem for a face recognition system, we propose a new PAD method based on hybrid features that combines information from both handcrafted and deep learning features. Our proposed method is novel in the following four ways.

- First, to the best of our knowledge, this is the first approach to PAD for face recognition systems using a combination of deep and handcrafted image features. By combining the deep and handcrafted image features, we enhance the detection accuracy compared to conventional state-of-the-art detection methods and reduce the variation in detection accuracy caused by the variation in face images.
- Second, instead of using multiple pre-trained CNN models for extracting image features as previous studies [34,35], we re-train a single CNN model using a large amount of real and presentation attack images for extracting deep features. Using this method, we make the CNN model more suitable for PAD problem for face recognition system and reduce the complexity of detection system compared to previous studies.
- Third, we use two methods for combining the detection results produced by using deep and handcrafted image features, including the score-level fusion and feature-level fusion. For the score-level fusion, the weight values for combining the deep and handcrafted images features are experimentally obtained to make them best describe the characteristics of the PAD problem for face recognition system.
- Finally, through [36], we made our trained CNN model with all the algorithms for PAD open to other researchers, to enable them to draw comparisons with our method.

Table 1 summarizes the comparison of previous research conducted on the PAD problem and our proposed method. In the rest of this paper, we will first describe our PAD approach for a face recognition system in detail in Section 2 using a combination of handcrafted and deep image features. Using the proposed method, we perform various experiments using two well-known public databases, NUAA [14] and CASIA [16], to evaluate the performance of our proposed method in comparison with previous methods. This will be described in Section 3. Based on the experimental results, we will give our explanations on the problem in Section 4. Finally, we will conclude our study in Section 5.

## 2. Proposed PAD Method for Face Recognition System

### 2.1. Overview of the Proposed Method

**Figure 1:**
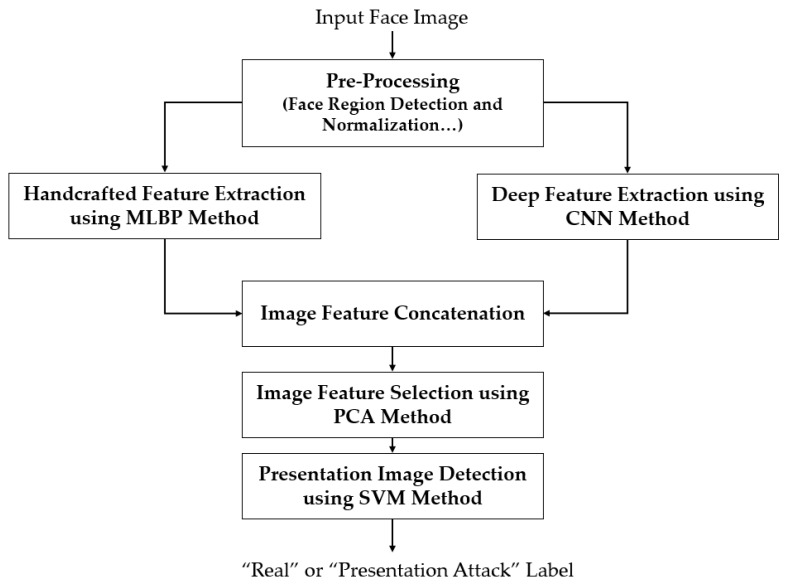
Flowchart of our proposed PAD method for a face recognition system.

Figure 1 shows an overview of our proposed method. Our proposed method uses a face image that is same as the input of a face recognition system as the input, and processes it to produce a binary decision of “real” or “presentation attack” image. The first step in our proposed method is to localize the face region in the input face image. For this purpose, we first pre-process the input image to extract the face region and compensate the in-plane rotation of the face if it exists. As a result, we obtain a frontal face region image that is sufficient to extract image features in subsequent steps. The pre-processing step is detailed in Section 2.2.

As explained in Section 1, our proposed method uses two feature extraction methods to extract the image features from the detected face region, including the handcrafted features (using MLBP method) and deep features (using CNN method). These feature extraction methods are detailed in Section 2.3 and Section 2.4, respectively. Using these two methods, we obtain two feature vectors which represent the characteristics of the input face images. To combine the information associated with the two feature vectors for the PAD problem, we further concatenate these feature vectors to form a combined feature vector, called hybrid feature vector. Using this vector, the input images are classified into “real” and “presentation attack” images using the PCA method for feature selection and SVM method for classification. The PCA and SVM methods are detailed in Section 2.5.

### 2.2. Face Region Detection and Normalization 

Input images for the PAD system can contain both face and background regions. Therefore, as explained in Section 2.1, our proposed method begins with the face region localization step in order to detect and extract the face region. As indicated by Benlamoudi et al. [20], the performance of the PAD method can be enhanced if the face region is well localized and normalized. Inspired by this result, we use a state-of-the-art face landmark detection method proposed by Kazemi et al. [37], called ensemble of regression trees (ERT), to detect the face region as well as 68 landmark points on the detected face. As a result, we can easily and efficiently detect the face region in the input image.

**Figure 2:**
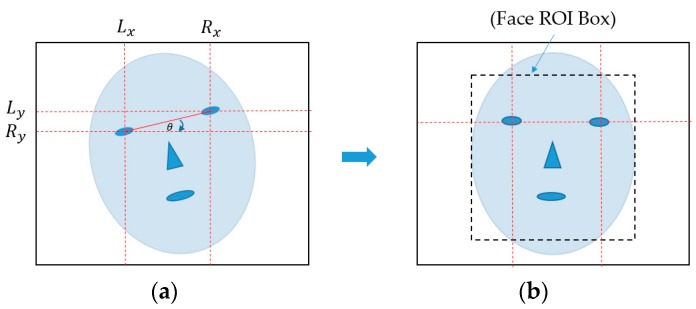
Description of in-plane rotation compensation and face region localization in our research: (**a**) input face image with in-plane rotation phenomenon and (**b**) resultant image of in-plane rotation compensation with localized face region box.

Normally, faces in input images can contain in-plane rotation because of the natural head pose of users during image acquisition. This phenomenon causes the misalignment problem between faces. As a result, the extracted image features are also misaligned between the face images, thus degrading the PAD performance. To address this misalignment problem, we further perform a face region normalization procedure by compensating the in-plane rotation using the detection results of the ERT method. In detail, we assume that (*L_x_*, *L_y_*) and (*R_x_*, *R_y_*) are the detected locations of the left and right eyes on the face, which are measured by averaging the corresponding landmark points around the left and right eyes, and (*C_x_*, *C_y_*) is the location of the center of face region measured by averaging all 68 landmark points on the face. Based on these points, in-plane rotation compensation is performed by rotating the entire face region around the center location of the face by an angle θ. The rotation angle θ is calculated in Equation (1), and the compensation procedure is illustrated in Figure 2:

(1) $$\theta =\tan^{-1}\left(\frac{R_{y}-L_{y}}{R_{x}-L_{x}}\right)$$

Using the in-plane rotation compensation method, the faces are aligned as shown in Figure 2b. Based on this result, we crop the detected face region to produce the face region image using the 68 landmark points and use this face region as the input of feature extraction methods.

### 2.3. Handcrafted Image Feature Extraction Based on MLBP Method

The previous PAD research for a face recognition system mainly used handcrafted feature extraction methods, such as Gabor filtering [15], local phase quantization [15], LBP [15,17,20], quality assessment [19], and DLTP [21], to extract image features. Among these feature extraction methods, the LBP method yielded the high detection accuracy. In our study, we use an extension of the LBP features, called MLBP features, for PAD for a face recognition system in order to enhance the detection ability of the LBP method. The LBP feature extraction method has been widely used to extract image features for many computer vision systems, such as face recognition [12], face expression recognition [38], finger-vein recognition [3], and human age estimation [39,40]. This method offers several advantages in extracted image features, including robustness to illumination and rotation variation [12,38,39,40]. An LBP operator is defined in Equation (2), where *R* and *P* indicate the radius of the circle and the number of surrounding pixels of the LBP operator, respectively; *g_c_* indicates the gray level of the center pixel of the circle; and *g_i_* indicates the gray level of the surrounding pixels:

(2) $${\mathrm{LBP}}_{R,P}=\sum_{i=0}^{P-1}s\left(g_{i}-g_{c}\right)2^{i}\;\mathrm{where}\;s\left(x\right)=\{\begin{array}{c} 1,\;if\;x\geq 0\\ 0,\;if\;x<0 \end{array}$$

Using the LBP method, we can extract a *p*-bit binary descriptor for each pixel in a given image using its surrounding pixels. As shown in Equation (2), the LBP method works as a local thresholding function for encoding the texture of an image in a small local region. Because of this reason, the LBP method offers illumination-invariant characteristic to the extracted image features, which plays a very important role in computer vision systems. The descriptor of each pixel obtained using the LBP method is used to describe the micro-texture in images, such as lines, spots, corners, and plane texture [39,40]. For our PAD research, we further classify the pixel descriptors into uniform and non-uniform descriptors, where the uniform descriptors are the ones having at most two bit-wise transitions from 0 to 1 (or from 1 to 0), while the non-uniform descriptors are those having more than two bitwise transitions from 0 to 1 (or from 1 to 0). As a result, the uniform descriptors mainly depict useful micro-textures (lines, spots, corners, and plane texture features), while the non-uniform descriptors depict very complex micro-textures and are normally originate from noise. To form the image features using the LBP method, we further accumulate the histogram of the uniform and non-uniform descriptors over an image and use this histogram as the extracted image features. Assuming that that the LBP operator has radius *R* and number of surrounding pixels *P*, the dimension of the extracted image features is given in Equation (3). By using different values of *R* and *P*, we can extract the LBP features at different scales (*R*) and resolutions (*P*):

(3) Dim=P×(P−1)+3

**Figure 3:**
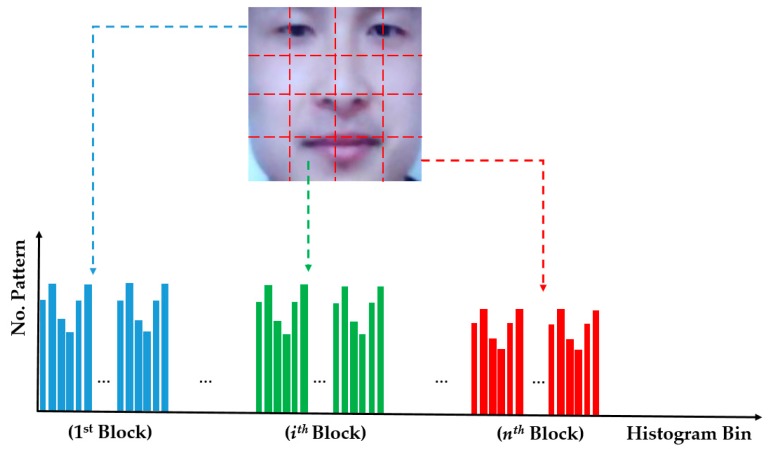
Description of image feature extraction using the MLBP method.

In a conventional setup, the image features are extracted by the LBP method by accumulating the histogram of the texture features over the entire face region. As a result, the extracted image features form a global feature vector and less affected by the misalignment problem. However, to obtain more powerful image features, the face region is then divided into several local regions. For each local region, a histogram feature vector is obtained using the conventional LBP method. Finally, the LBP features of the entire image are obtained by concatenating the feature vectors of all local regions. Figure 3 illustrates the image feature extraction using the LBP method. Because this feature vector is obtained using a single pair of radius (*R*) and resolution (*P*), we call it “single-level LBP” here. Based on Equation (3), the number of components of the single-level LBP features is equal to “*M × N × Dim*” where *M* and *N* are the numbers of local regions in the horizontal and vertical directions, respectively.

Although the single-level LBP features are efficient for PAD for a face recognition system [20], the use of a single scale and resolution pair is its limitation because face images contain considerable variation. Thus, to capture richer information from a face image, we use multi-level LBP (MLBP) features instead of single-level LBP features. In detail, we concatenate several single-level LBP features, which have different values of radius (*R*) and resolution (*P*). As a result, the MLBP features contain texture information at various scales and resolutions. In our experiments, we divide the face region into 2-by-2 local regions and extract the MLBP features using three values of radius (*R* = 1, 2, and 3) and three values of resolution (*P* = 8, 12, and 16). For a special case of *R* = 1, we only use *P* = 8 (the basic LBP operator). As a result, we obtain a feature vector of 3732-components for each face image.

### 2.4. Deep Image Feature Extraction Based on CNN Method

Although the handcrafted image feature extraction methods have been proven to be sufficient for PAD for a face recognition system, their performances depend on the characteristics of the presentation attack images. This is because the handcrafted feature extraction methods are designed based on expert knowledge of the designer on the problem. As a result, they reflect limited aspects of the problem. To extract more efficient features for the PAD problem, we further use a learning-based technique using the CNN method to learn a feature extraction model. As proven by many previous studies, CNN is a powerful method and has been successfully applied to many computer vision systems such as for image classification [24,25,26,27], gender recognition [32], face-based human age estimation [30,31], and PAD for finger-vein recognition system [9] as well as iris, face, and fingerprint recognition systems [10]. As shown by Menotti et al. [10], the CNN method can be an alternative for detecting presentation attack images.

**Figure 4:**
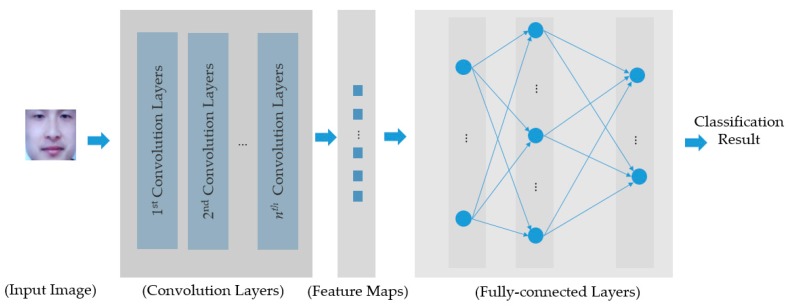
Description of general structure of a CNN.

Figure 4 shows the general structure of a CNN, where a CNN comprises two key parts: convolution layers and fully-connected layers. The convolution layers are responsible for performing the image manipulation processes using the convolution operations to manipulate and extract the image features. The filter coefficients are obtained automatically by using the training process, and are dependent on the characteristics of the input training images. Each convolution layer can be followed by a cross-channel normalization layer, a rectified linear unit (ReLU), and a pooling layer to transform the convolution operation results and make the CNN invariant to image translation and illumination. As a result, we can extract several feature maps (marked as “Feature Maps” in Figure 4), using which the CNN classifies the input images into pre-defined categories using fully-connected layers.

In the present study, we construct our CNN, for extracting deep image features for the PAD problem, based on a very deep CNN architecture proposed by Simonyan et al. [25], called VGG Net-19 network. For the PAD problem for a face recognition system, there are only two classes: “real” and “presentation attack” images. Therefore, we change the number of output neurons in the original VGG Net-19 network from 1000 to 2. Table 2 shows, in detail, the CNN network used in our study. By training the network in Table 2 using a large volume of training data, we can obtain a CNN model for classifying images into real and presentation attack classes. Then, using the trained CNN model, we can extract a 4096-component image feature vector using the second fully connected layer (fc7 in Table 2), and use this feature vector to detect presentation attack images using the SVM method.

To train the CNN in our study, we use a well-known gradient descent method, called stochastic gradient descent (SGD), with momentum method [24]. In a conventional gradient descent method, the network parameters are updated when all training data are passed through the network. Therefore, it is difficult to train the model using a large amount of training data. However, by using SGD with the momentum method, the network parameters are updated every time a small amount of training data (equal to mini-batch size) passes through the network. As a result, training with a large amount of training data can be successfully achieved with faster convergence. The SGD training method comprises various parameters including momentum, learning rate, and mini-batch size etc. The detail values of these parameters used in our experiments are explained, which are detailed in Section 3.

Although the CNN method is sufficient for many image-based systems, it faces the over-fitting problem caused by the use of a large volume of network parameters [9,24,41]. For example, using the CNN shown in Table 2, the training process must learn about 140 million parameters. As a result, the training process requires a large volume of training data to successfully train its parameters. However, such a large volume is difficult to collect. To reduce the effects of the over-fitting problem, we use three common methods: dropout, data augmentation, and transfer learning [9,24,41]. In the first method, we disconnect several connections between the neurons in a fully connected layer with a probability of dropout value (from 0 to 1) [41]. In the second method, we generalize the training data by artificially creating additional images from each image in the training data. This procedure helps to significantly increase the training data volume as well as generalize the training data. Finally, we apply the transfer learning method during network initialization to well initialize the network parameters using a pre-trained network that was successfully trained using a very large volume of training data [9].

### 2.5. Feature Selection Using PCA and Classification using SVM Method

Using MLBP and CNN methods, we can extract the two feature vectors which describe the characteristics of a face image. To create a feature vector that combines the information from each single vector, we further concatenate the two feature vectors to form a hybrid feature vector. Because the hybrid feature vector is a combination of two types of feature vectors, it contains richer information for the PAD problem than a single feature vector. For convenience, we represent the handcrafted feature vector as *f_h_* and the deep feature vector as *f_d_*. Then, the hybrid feature vector is formed by concatenating the two vectors as shown in Equation (4):

(4) $$f=\left[f_{h},f_{d}\right]$$

However, the hybrid feature vector has increased the dimensionality of image features. In detail, we extract a 3732-component feature vector *f_h_* by using the MLBP method and a 4096-component feature vector *f_d_* using the CNN method. As a result, the hybrid feature vector is a vector in 7828-dimensional space (3732 + 4096). The high dimensionality of the feature vector requires high processing power and a very complex SVM classifier for classification. To address this problem, the subspace method has been widely used [9,12,20,32]. Thus, our proposed method uses the subspace method to reduce the dimensionality of the hybrid feature vector, as shown in Figure 1. For this purpose, we invoke the PCA method to select a smaller number of components for the hybrid feature vector before classifying the real and presentation attack images using the SVM method. By using the PCA method, we can arbitrarily select a small number of principal components, which have the largest variation, from the original number of components. As a result, the feature vector with this small number of principal components can have enough power to describe the original features while having much smaller dimension than that of the original features. In our experiments, the number of principal components is experimentally selected by which the best classification accuracy is obtained.

With the selected features using PCA method, our proposed method uses SVM to classify the input images into real and presentation attack images. By definition, the SVM method is used to find the best hyper-plane that can separate the samples of one class from those of other classes using several support vectors. For a nonlinear problem, the SVM method uses various kernel functions to map the input feature vectors to a higher-dimensional space in which the problem can be linearly separated. To classify an input feature vector, the SVM evaluates the sign of a function as shown in Equation (5). In this equation, the SVM uses *k*-support vectors with the model parameters of $a_{i}$ and b, and $K\left(x_{i},x_{j}\right)$ is the kernel function [42]. These parameters are trained and stored in a trained SVM model using training data. In our experiments, we use three types of SVM kernels—linear kernel, radial basic function (RBF) kernel, and polynomial kernel—to measure the accuracy of our PAD method, as shown in Equations (6)–(8) [9,42,43]. In addition, we use MATLAB environment for CNN, PCA, and SVM implementation [44,45,46]:

(5) $$f\left(x\right)=sign(\overset{k}{\underset{i=1}{\sum }}a_{i}y_{i}K\left(x,x_{i}\right)+b)$$

(6) $$\mathrm{Linear}\;\mathrm{kernel}:\;K\left(x_{i},x_{j}\right)=x_{i}{}^{T}x_{j}$$

(7) $$\mathrm{RBF}\;\mathrm{kernel}:\;K\left(x_{i},x_{j}\right)=e^{-\gamma \left\|x_{i}-x_{j}\right\|{}^{2}}$$

(8) $$\mathrm{Polynomial}\;\mathrm{kernel}:\;K\left(x_{i},x_{j}\right)={\left(\gamma x_{i}{}^{T}x_{j}+coef\right)}^{degree}$$

## 3. Experimental Results

### 3.1. Databases and Performance Measurement Criteria

**Figure 5:**
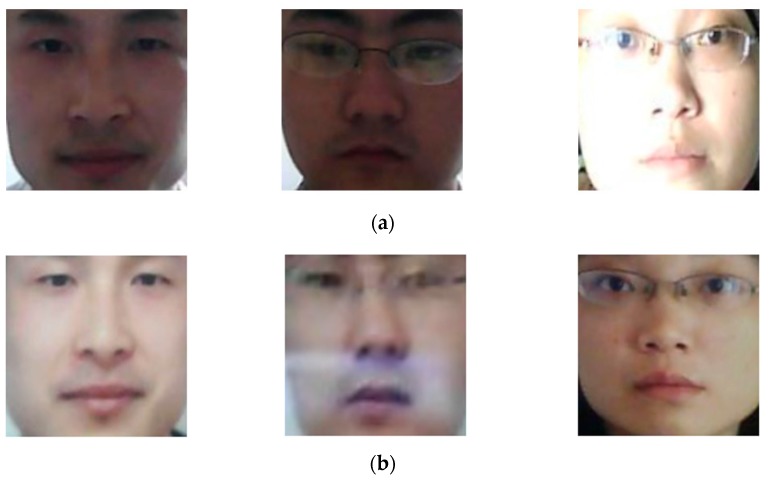
Example face region images in NUAA database: (**a**) real images and (**b**) presentation attack images.

To the best of our knowledge, the NUAA database is one of the first public databases for training and evaluating the performance of the PAD method for a face recognition system [14]. This database simulates a simple and general method that re-captures a printed photograph of users for attacking a face recognition system. The NUAA database contains real and presentation attack face images of 15 persons. For each person, they captured both real and presentation attack images in three different sessions using generic cheap webcams and real face and printed photograph of users. The photographs were either printed on photographic paper or 70 g A4 paper [14]. Thus, the NUAA database contains 5105 real and 7509 presentation attack face images in color space with 640 × 480 pixels of image resolution. In this database, using the collected images, the training and testing sub-databases are predefined for training and testing of the PAD method, through which the performances of various PAD methods can be compared. In detail, the training database contains 1743 real and 1748 presentation attack face images, while the testing database contains 3362 real and 5761 presentation attack face images. In addition, as explained in Section 2.4, the CNN method requires a large volume of training data to reduce the effects of the over-fitting problem. Therefore, we enlarge the training database by artificially creating additional images from the original ones by shifting, cropping, and scaling methods. In detail, we create 24 additional images by shifting, cropping, and scaling each original face image in both horizontal and vertical directions. As a result, we obtain a total of 25 images (one original image and 24 artificial images) for each original image in the training database. To compare the detection performance of our proposed method with those of the previous methods, we apply this procedure to only the training database, and not the testing database. The NUAA database and its training and testing sub-databases are detailed in Table 3. In addition, Figure 5 shows some example face region images, which resulted from the application of the face detection method in the NUAA database.

**Figure 6:**
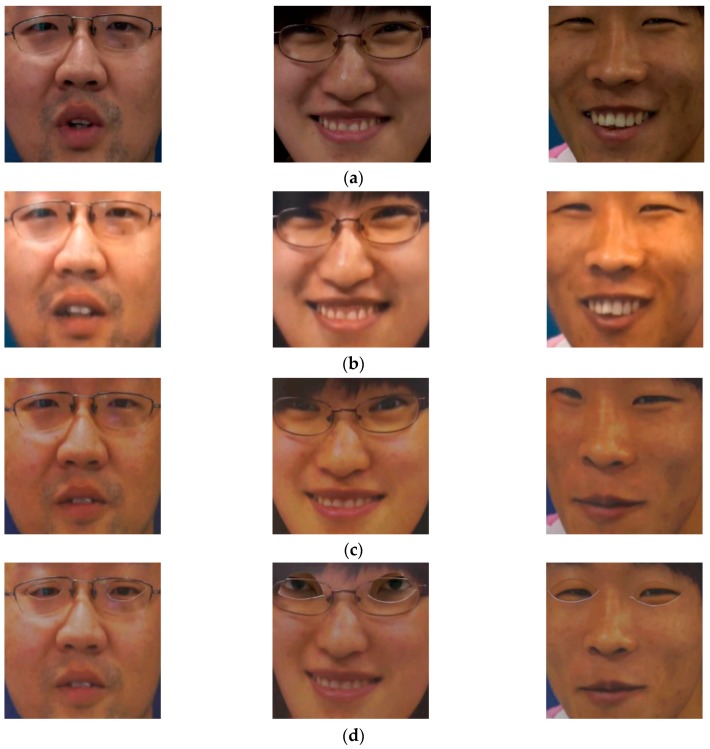
Example face region images from CASIA database: (**a**) real access images, (**b**) presentation attack images using video display, (**c**) presentation attack images using wrap photo, and (**d**) presentation attack images using cut photo.

Since the images in NUAA database were captured using cheap webcams, its quality is limited. To evaluate the performance of our proposed method in various attack scenarios, we use another public database, called CASIA database [16]. The CASIA database contains real and presentation attack face images of 50 persons, which is much larger than the number of clients used in the NUAA database. In addition, the CASIA database contains larger variation in quality of face regions (low quality, normal quality, and high quality) and the attacking methods (using wrap photo, cut photo, and video display). For each person, the database consists of 12 video clips captured in three categories of face region quality and three attack methods. Similar to the NUAA database, the training and testing sub-databases in the CASIA database are predefined. In details, real and presentation attack data from 20 persons are assigned as the training data and the remaining data of 30 persons are assigned as the testing data. Using the face detection method, we detect face region images for training and testing sub-databases, as shown in Table 4. As shown in this table, we collect 45,052 face images from the training database and 65,662 images from the testing database. Similar to the experiments conducted with the NUAA database, we created artificial images for the training database in order to generalize the training database and reduce the effects of the over-fitting problem. For this purpose, we created two images from each original image in the training database to increase the number of training images from 45,052 to 90,104 images while keeping the number of images in the testing database constant. In Figure 6, we show some example face region imageTs from the CASIA database according to the attacking methods of using video display, wrap photo, and cut photo.

For evaluating the performance of a PAD method, we use two metrics: the attack presentation classification error rate (APCER) and the bona fide (real) presentation classification error rate (BPCER) [9,47,48]. By definition, APCER indicates the proportion of attack presentations incorrectly classified as bona fide presentations, while BPCER indicates the proportion of bona fide (real) images incorrectly classified as presentation attack images. APCER and BPCER are analogous to the false acceptance rate (FAR), and false rejection rate (FRR) in a conventional recognition system, respectively. In addition, we use the average classification error rate (ACER) to measure the average classification error, as shown in Equation (9). The PAD method with a lower measured value of ACER indicates a better detection performance of the system:

(9) $$\mathrm{ACER}=\frac{\mathrm{APCER}+\mathrm{BPCER}}{2}$$

In our experiments, for each database (NUAA or CASIA database), we use the training database to train the CNN model for deep feature extraction, the PCA transformation matrix, and an SVM classifier for real and presentation attack classification. With the result of the training process, we use the testing database to measure the performance (in terms of APCER, BPCER, and ACER) of the PAD method.

### 3.2. Experimental Results

#### 3.2.1. Detection Accuracy of PAD Method Using Only Handcrafted Features

In our first experiment, we measure the detection accuracy of the PAD method that uses only MLBP features for detection problem. This experiment aims to evaluate the detection ability of MLBP features for the PAD problem. For this purpose, the hybrid features in Figure 1 are replaced by MLBP features while keeping all other processing steps same. In addition, we measure the detection accuracy of the PAD method in both cases of with and without applying PCA for feature selection to validate the efficiency of the PCA method for feature dimensionality reduction. The detailed experimental results using the NUAA and CASIA databases (which were described in Table 3 and Table 4) are given in Table 5. In this table, we also report the selected number of principal component (denoted as “No. PC”) by which the best detection accuracy is obtained in our experiments.

**Figure 7:**
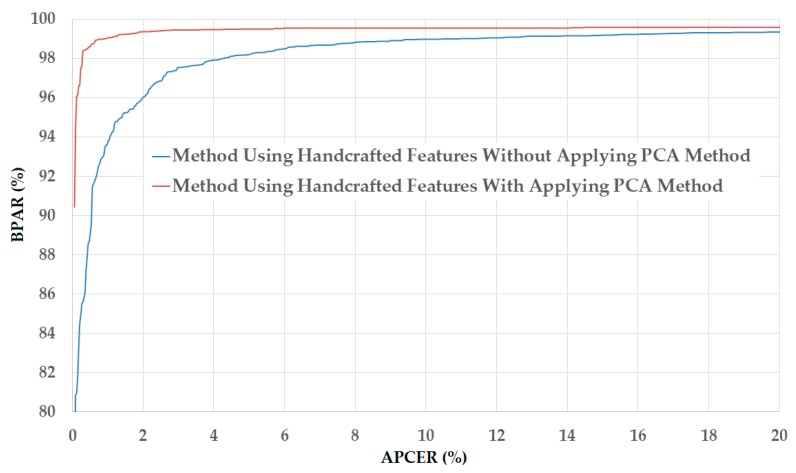
DET curves of the detection system that uses only handcrafted image features on NUAA database with and without applying PCA method.

The upper part of Table 5 shows the experimental results obtained using the NUAA database. As shown in this table, we obtain the smallest detection error (ACER) of 2.492% using the raw MLBP features and linear kernel of the SVM method. However, using the PCA method, the detection errors are further reduced to 2.077%, 0.966%, and 0.667% using linear, RBF, and polynomial kernels of the SVM method, respectively. These experimental results indicate that the PCA method is sufficient for reducing the dimensionality of the image features and enhancing the detection accuracy of the PAD method using handcrafted image features on the NUAA database. Benlamoudi et al. [20] and Parveen et al. [21] used the LBP and DLTP features, respectively, on the NUAA database, and obtained smallest errors of 1.00% using LBP features and 3.5% using DLTP features. A comparison of their detection errors with our results in this experiment shows that the PAD method based on MLBP features outperforms that based on LBP or DLTP features in the case of using the NUAA database. Figure 7 shows the detection error tradeoff (DET) curves of the PAD method using handcrafted image features in two cases of with and without applying the PCA method. This figure plots the changes in APCER as a function of the bona-fide presentation acceptance rate (BPAR), which is measured as (100-BPCER) (%). In addition, we draw two curves corresponding to the best detection accuracies with ACER of 2.492% and 0.667% for the cases of without and with applying the PCA method, respectively. This figure confirms that the PCA method is sufficient for reducing the dimensionality of image features and enhancing the detection accuracy of the PAD method in case of using the NUAA database.

The lower part of Table 5 shows the experimental results of the PAD method using handcrafted image features on the CASIA database in Table 4. As shown in Table 5, the smallest detection errors (ACERs) obtained in these experiments are 10.504% using the RBF kernel and 10.566% using the polynomial kernel for the cases of without and with applying the PCA method, respectively. As indicated by these experimental results, the error rate produced by the system using the PCA method is slightly larger than that produced without applying the PCA method. However, these two errors are almost similar (the difference is just about 0.062%) and the use of PCA helps to significantly reduce the dimensionality of the original feature vector.

**Figure 8:**
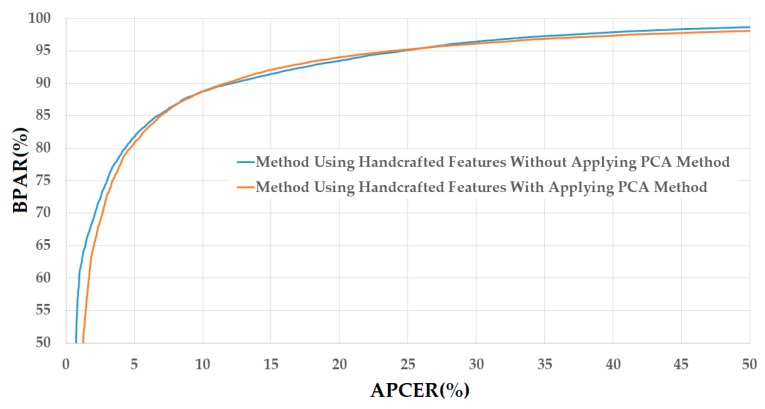
DET curves of the detection system that uses only handcrafted image features on CASIA database with and without PCA applying method.

Figure 8 shows the DET curves of these experiments corresponding to these two best detection accuracies. As shown in this figure, the two DET curves are almost overlapped. Therefore, we can conclude that the PCA method is also reasonable for the PAD method. Compared to the detection errors generated in the case of using the NUAA database, those generated in the case of using the CASIA database are much larger. This result is caused by the fact that the two databases are different, and thus, have different characteristics of real and presentation attack images. In addition, these results demonstrate that the handcrafted image features have large variation in detection accuracy depending on the characteristics of the database. Thus, this problem can reduce the reliability of the detection system that uses only handcrafted image features.

#### 3.2.2. Detection Accuracy of PAD Method Using Only Deep Features

**Figure 9:**
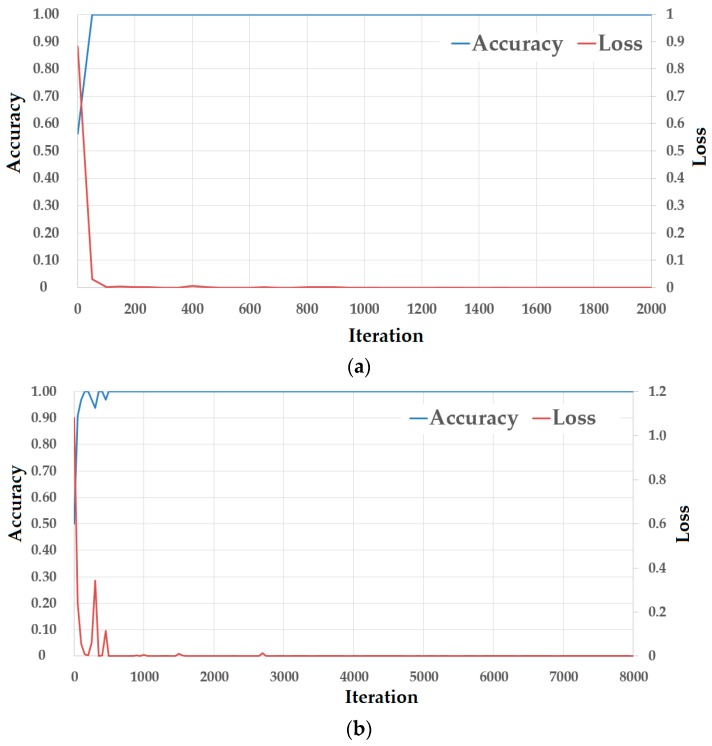
Loss curves of training procedures using (**a**) NUAA database, and (**b**) CASIA database.

We next perform experiments to measure the detection accuracy of the PAD method that uses only deep features. As the first experiment in this section, we perform a training procedure to train CNN models (network structure described in Table 2) using the SGD algorithm on NUAA and CASIA databases. Table 6 shows the parameters for the SGD algorithm. As explained in Section 2.3, we apply the transfer learning method to reduce the effects of the over-fitting problem during the training process. For implementation, we use a pre-trained model that was successfully trained using ImageNet database [24] and VGG Net-19 network model [25], to initialize the parameters of our CNN model described in Table 2. Because of using the transfer learning technique, the parameters of our CNN model are well-initialized and the consequent training process shows rapid convergence. Therefore, as shown in Table 6, we only use a small initial learning rate (0.001) and few training epochs (6 epochs) for our training procedure. By definition, an epoch is a unit that indicates that all training data are passed through the network. The learning rate is then dropped by a factor of 0.1 every two epochs to fine-tune the network parameters. The results of the training procedures in the cases of using the NUAA and CASIA databases are given in Figure 9a,b, respectively.

In this figure, the horizontal axis represents “iteration” which indicates the number of times a block of training images with “mini-batch size” is passed through a network and the network parameters are updated. As shown in Table 6, we set the mini-batch size as 32, which means that the network parameters are updated every time a block of 32 images is passed though the network. As shown in Figure 9, the training procedures are successfully conducted, with the loss approaching zero and the training accuracy approaching 100% after several hundred iterations.

**Figure 10:**
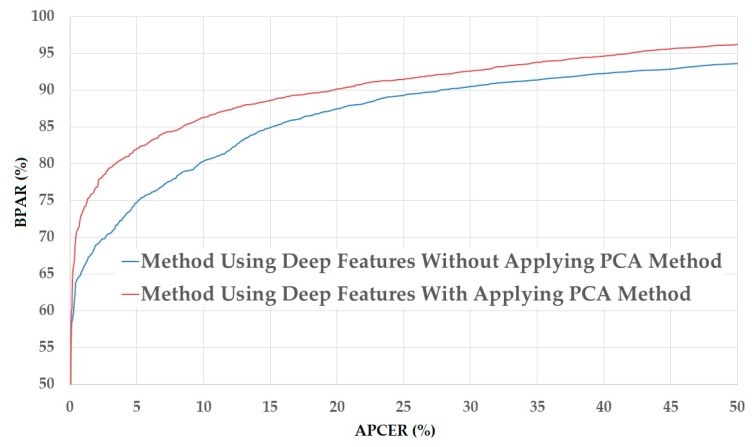
DET curves of the detection system that uses only deep image features on NUAA database with and without applying PCA method.

**Figure 11:**
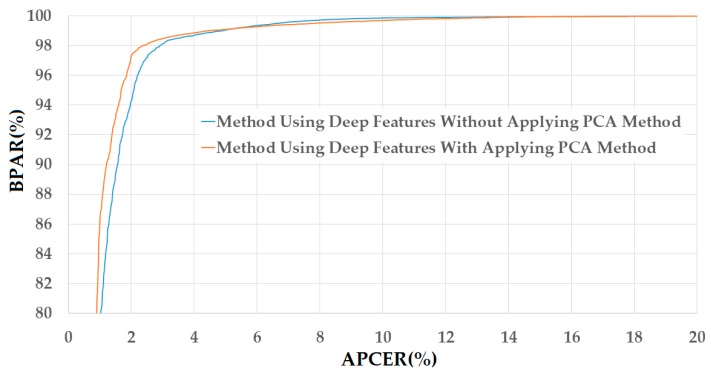
DET curves of the detection system that uses only deep image features on CASIA database with and without applying PCA method.

Using the results of training the CNN models, we extract the image features and perform “real” and “presentation attack” classification as explained in Section 2.4 and Section 2.5. The detailed experimental results of this experiment are given in Table 7, where using the deep features without applying the PCA method, we obtain an error rate of 14.609% using the polynomial kernel of the SVM method and the NUAA database. By applying the PCA method on deep features, we further reduce the detection error to 11.247% using the linear kernel of the SVM method. In the case of using the CASIA database, we obtain the smallest error (ACER) of 2.398% using the linear kernel of SVM on original deep features (without applying PCA method) and 2.174% using PCA and the polynomial kernel of SVM method. Figure 10 and Figure 11 show the DET curves of these experiments. The experimental results indicate that the deep features are also sufficient for the PAD method. In addition, the PCA method is sufficient for not only reducing the dimensionality of the image features but also enhancing the detection accuracy of the PAD method that uses deep features only.

As shown in Table 5 and Table 7, in the case of using the NUAA database, the detection error generated by the PAD method that uses only the deep features is worse than that generated by the PAD method that uses only handcrafted features (ACER of 0.667% in Table 5 versus ACER of 11.247% in Table 7). However, in the case of using the CASIA database, the detection error generated by the PAD method that uses only deep features is much better than that generated by the PAD method that uses only handcrafted features (ACER of 10.566% in Table 5 versus ACER of 2.174% in Table 7). These results show that the performances of the PAD methods that use only handcrafted image features or deep features varies significantly depending on the database used. Consequently, the reliability of such systems is low.

#### 3.2.3. Detection Accuracy of our Proposed PAD Method

The detection results in Section 3.2.1 and Section 3.2.2 show that handcrafted and deep features are sufficient for detecting presentation attack images in a face recognition system. In our next experiment, we evaluate the detection performance of our proposed method, which uses hybrid features instead of using only handcrafted or only deep features (as depicted in Figure 1). Similar to our experiments in Section 3.2.1 and Section 3.2.2, we measure the detection accuracy in two cases of with and without applying the PCA method and using three types of SVM kernels (linear, RBF, and polynomial). The detailed experimental results using both NUAA and CASIA databases are given in Table 8.

**Figure 12:**
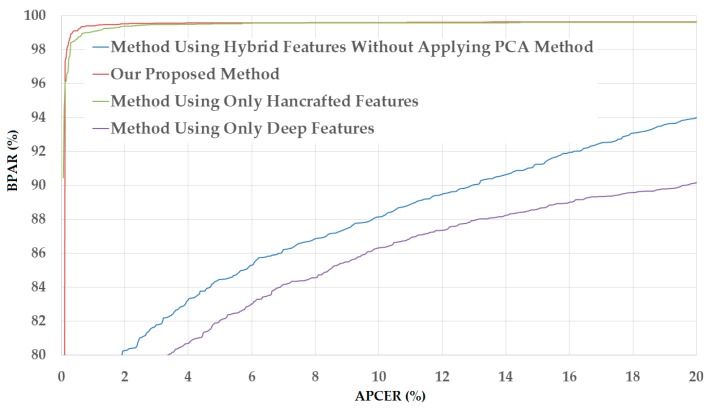
DET curves of our proposed PAD method in comparison with other methods on NUAA database.

The upper part of Table 8 shows the experimental results of our proposed PAD method in the case of using the NUAA database. As shown in this table, we obtained the smallest detection error (ACER) of 10.077% using the linear kernel of SVM and without applying the PCA method. However, we obtain a much smaller error (ACER) of 0.456% by applying PCA method on the hybrid image features and using the polynomial kernel of SVM. This result again confirms that the PCA method is sufficient for enhancing the detection accuracy in our proposed method. This detection error is smaller than those generated by the PAD method that only uses handcrafted features (0.667% in Table 5) or only uses deep features (11.247% in Table 7). In addition to the data shown in Table 8, we show the DET curves for various PAD method configurations in Figure 12, including the PAD method that uses only handcrafted image features, the PAD method that uses only deep features, our proposed PAD method that uses hybrid features without applying the PCA method, and our proposed PAD method. As we can observe from this figure and data in Table 8, our proposed method outperforms the other three PAD method configurations.

The lower part of Table 8 shows the detection errors of our proposed PAD method in the case of using the CASIA database (as described in Table 4). As shown in this table, we obtain the smallest error (ACER) of 2.189% using the RBF kernel of SVM and the raw hybrid features (without PCA application). This error is then reduced to 1.696% using the linear kernel of SVM and applying the PCA method on the hybrid features (our proposed method). A comparison of the detection errors of this experiment with those in Section 3.2.1 and Section 3.2.2 shows that the error generated by our proposed method (ACER of 1.696%) is smaller than that generated by the PAD method that uses only handcrafted features (10.566% in Table 5) or only deep features (2.174% in Table 7).

As suggested by previous studies [34,35], the deep and handcrafted image features can be also combined using another fusion method, called score-level fusion. Inspired by this suggestion, we also consider this fusion method to measure the detection accuracy of PAD system and compared with our proposed method that uses feature-level fusion approach. In detail, the deep and handcrafted image features are respectively used as the inputs of two SVMs to classify input images into either real or presentation attack image, as shown in Section 3.2.1 and Section 3.2.2. Consequently, we can obtain two score values from the two SVMs, which stand for the probabilities of an input image is classified as a real or presentation attack image. These scores are then combined using weighted sum rule to make the final decision of which class the input image belongs to as shown in Equation (10). In this equation, the *w*_1_ and *w*_2_ are the weight values of CNN and MLBP methods, respectively; and the *S*_1_ and *S*_2_ are the prediction scores of PAD systems that use only CNN or only MLBP features, respectively:

(10) $$S=w_{1}S_{1}+w_{2}S_{2}$$

In our experiments, the optimal weight values are obtained experimentally instead of using fixed values in previous studies [34,35]. As shown in Table 9, we obtained the smallest detection errors of 0.630% using NUAA database (with *w*_1_ = 0.15 and *w*_2_ = 0.85) and 1.792% using CASIA database (with *w*_1_ = 0.75 and *w*_2_ = 0.25). These errors are little higher than those produced by the use of feature-level fusion method for combination in our method (0.456% using NUAA database and 1.696% using CASIA database in Table 8). From these experimental results, we find that the feature-level fusion method is more suitable than score-level fusion method for our problem.

**Figure 13:**
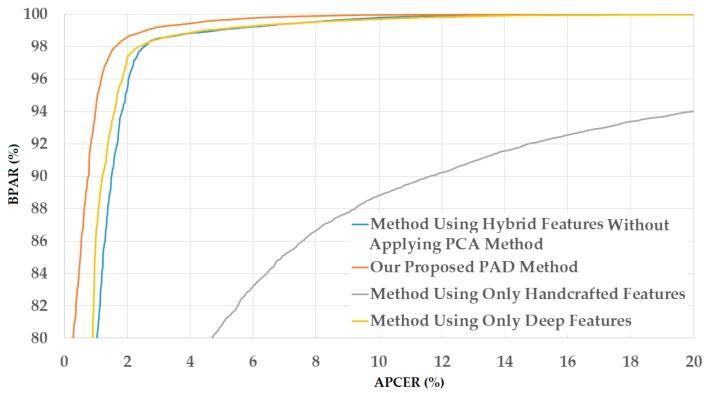
DET curves of our proposed PAD method in comparison with other methods on CASIA database.

For demonstration, we show the DET curves of this experiment in the case of using the CASIA database in Figure 13. Through the detection accuracy in Table 8 and Table 9, and the DET curves in Figure 12 and Figure 13, we conclude that our proposed method is sufficient for reducing the detection error of the PAD method that uses single feature extraction (only handcrafted image features or only deep features). In addition, we confirm that the PCA method is sufficient for reducing the dimensionality of the image features and the detection error in our proposed method.

To validate the efficiency of our proposed method for solving the PAD problem, we further perform a comparison of the detection performances between our proposed method and previous research using the same testing databases (NUAA and CASIA). As explained in Section 3.1, the NUAA and CASIA database are the public databases and they have been widely used in previous research on the PAD method for face recognition system [14,15,16,17,20,21,23]. In addition, these databases were provided with pre-defined training and testing dataset. As a result, we can have a fair comparison with previous studies. The detailed comparison is given in Table 10 and Table 11 for the NUAA and CASIA databases, respectively. In the case of the NUAA database, the baseline method proposed by the author of the database gave an error of about 9.5% [14]. Later, Maatta et al. [15] used the Gabor filters, LPQ method, and LBP method for extracting image features, and reported the errors to be about 9.5% for Gabor filters, 4.6% for LPQ, and 2.9% for LBP. Benlamoudi et al. [20] used LBP features in combination with the Fisher score for feature selection and SVM for classification, and reported an error of about 1.00% on in the case of using the NUAA database. Parveen et al. [21] used the DLTP method for extracting image features and SVM for classification, and reported a detection error of 3.5%, which was lower than that reported for the Gabor or LPQ method, but still higher than that reported for the LBP method. Comparing these detection performances, we can see that our proposed PAD method significantly outperforms all previously proposed methods by producing the lowest errors (ACER of 0.456%).

In the case of using the CASIA database, an error rate (ACER) of about 17.0% was obtained using the baseline method proposed by the author of the database [16] using DoG image features and SVM for classification. The error was then reduced to 13.1% by Benlamoudi et al. [20], LBP method for image feature extraction, Fisher score for feature selection, and SVM for classification. To extract richer information from the face region image, Boulkenafet et al. [17] applied the LBP feature extraction method on three channels of color images in YCbCr color space and used SVM for classification. Because of using color information, they could reduce the error to about 6.2%. Parveen et al. [21] used the DLTP method, instead of the LBP method, for feature extraction and obtained an error of 5.4% using the CASIA database. Akhtar et al. [23] extracted the information from image patches for discriminating the real and presentation attack images. Consequently, they obtained an error rate of 5.07%. As shown in the experimental results in Table 8, our proposed PAD method offers an error rate (ACER) of 1.696%, which is much smaller than all of the previously reported errors. From the comparisons in Table 10 and Table 11, we can conclude that our proposed method is sufficient for solving the PAD problem and outperforms previous research.

As the final experiment in this Section, we measured the processing time of our proposed PAD method. For this experiment, we used a desktop computer equipped with an Intel Core i7 CPU (3.4 GHz) with 64 GB of RAM memory, and a TitanX graphics processing unit (GPU) [49] for running CNN model. The detailed experimental results are given in Table 12. As shown in this table, our proposed method takes about 50.5 milliseconds (ms) to process one input face image. Based on this result, we can conclude that our proposed method can process at the speed of about 20 frames per second (1000/50.5).

#### 3.2.4. Detection Accuracy of our Proposed PAD Method According to Characteristics of Images

In this section, we further investigate the effects of image quality and attacking methods on the performance of the PAD method. Since the NUAA database was collected by re-capturing printed photographs of users without any additional information on image quality, we do not use this database in the experiments presented in this section. In contrast, the CASIA database is a more complex database, and was collected by simulating several attacking methods; moreover, it uses three categories of face region quality during image acquisition. As a result, in our experiments, we separate the CASIA database into six sub-databases according to face region quality and attacking methods. For the experiments presented in this section, we apply our proposed PAD method (as shown in Figure 1) on these six sub-databases of CASIA database to measure the detection performance according to the characteristic of input images, i.e., image quality and attacking methods.

In the first experiment, based on the quality of face regions in video clips provided by the author of CASIA database, we separate the entire database into three sub-databases of “Low Quality Database”, “Normal Quality Database”, and “High Quality Database” as shown in Table 13. For these databases, we only use the real and presentation attack data that are in same quality category. Based on this criterion, we obtain the three quality databases, as detailed in Table 13, using the face detection method explained in Section 2.2. To reduce the effects of the over-fitting problem, we also perform data augmentation on the training data of these quality-based databases to generalize the training data while keeping the testing data same as the original data, for comparison with previous research. The detailed experimental results are given in Table 14. As shown in this table, we obtain the smallest detection error (ACER) of 1.834% using the “Low Quality Database” and the polynomial kernel of SVM, an error of 3.950% using the “Normal Quality Database” and the RBF kernel of SVM, and 2.210% using the “High Quality Database” and RBF kernel of SVM. These experimental results are quite different and they indicate that the quality of the face region is an important factor for detecting presentation attack images using our proposed PAD method.

**Figure 14:**
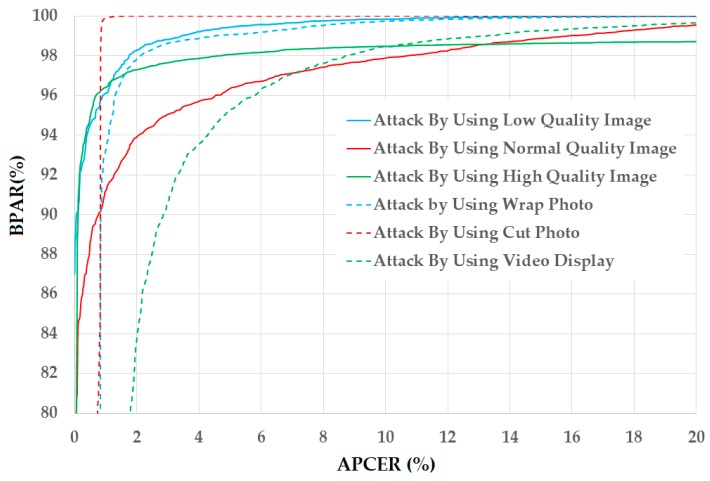
DET curves of the detection system using our proposed PAD method on six sub-databases of CASIA database according to image quality (low, normal, and high quality) and attacking methods (using wrap photo, cut photo, and video display).

In the second experiment, we divide the entire CASIA database into three sub-databases—“Wrap Photo Database”, “Cut Photo Database” and “Video Display Database”—according to the three attacking methods. For these databases, the real data include all real data in the CASIA database and the presentation attack data are selected based on the attacking method. Consequently, we obtain the three databases for this experiment as given in Table 15. Finally, we obtain experimental results as shown in Table 16, where the errors (ACERs) of 2.054%, 0.545%, and 4.835% are obtained for the databases of “Wrap Photo Database”, “Cut Photo Database”, and “Video Display Database”, respectively. As indicated in these experimental results, the error generated using video display to attack a face recognition system is the largest among the three attacking methods. This shows that it is most difficult to detect the presentation attack images that are developed using video display of the face as compared to the other two attacking methods, probably because the presentation attack images developed using a video display have less negative effects, such as blur, noise, or additional illumination, than those using wrap or cut photo. As a result, the presentation attack images developed using a video display look more similar to real images than those developed using the other two methods. For demonstration, we show the DET curves of all experiments in this section in Figure 14, where the characteristics of the presentation attack images (quality and attacking methods) strongly affect the PAD method by yielding very different detection accuracies according to types of presentation attack images.

Finally, we compare the detection accuracy yielded by our proposed method with those obtained in various previous studies using the same presentation attack databases according to image quality and attacking methods. The detailed comparisons are given in Table 17. As shown in this table, our proposed method outperforms all previous methods by yielding the lowest detection error.

## 4. Discussion

**Figure 15:**
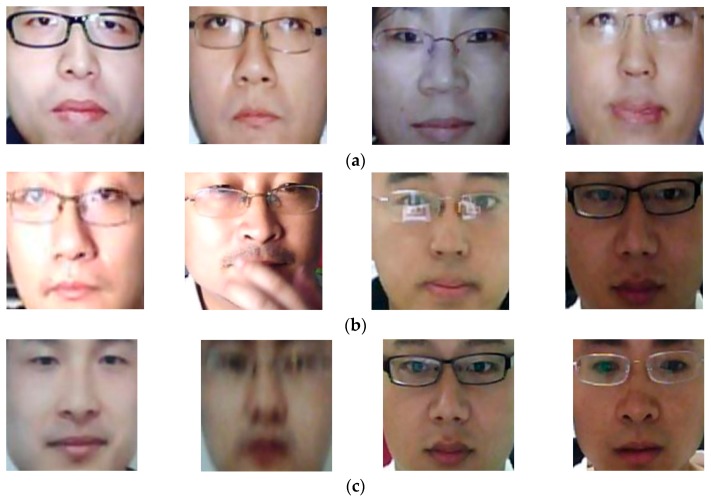
Examples of detection result images by PAD method that uses only MLBP features for detection problem on NUAA database: (**a**) “presentation attack to real” error cases; (**b**) “real to presentation attack” error cases; and (**c**) correct detection cases.

Figure 15 shows some examples of the resultant images of the PAD method that uses only handcrafted (MLBP) features on the NUAA database. This figure shows images for three cases: “presentation attack to real” classification error (Figure 15a), “real to presentation attack” classification error (Figure 15b), and correct classification (Figure 15c). As observed from this figure, the error cases mainly occur when the presentation attack images are clear and of good quality (Figure 15a) or the real images have blurred effects or large illumination variation (Figure 15b). In a conventional face recognition system, the input face images are directly captured from real 3-D faces of users. Therefore, the quality of the captured face images is very high. On the other hand, since the presentation attack images are re-captured using photo/video displays that are in 2-D space, the consequent presentation attack images can contain blur or plane texture features. However, there are some cases in which the real images are captured under poor capturing conditions, such as high illumination or vibration of camera (or face) during capturing, or the presentation attack images are captured under a focused condition using high-quality photo/video displays. As a result, the appearance of real images become little blurred and/or white, as shown in Figure 15b, while that of presentation attack images becomes more distinctive, as shown in Figure 15a.

Because the MLBP features are used to measure the skin details on face region, such as edges, corners, and blobs, the good-quality presentation attack images can produce MLBP features that are similar to those produced using slightly poor real images. Consequently, the PAD method using MLBP features produces errors. For demonstration, we show some correct detection result images of the PAD method using MLBP features in Figure 15c. In this figure, the first two images (from left to right) are the presentation attack images that contain blurred and unclear appearance, and are correctly detected as the presentation attack images. In the last two images, the PAD method correctly classifies them as real images because of their high quality and distinctive appearance. Our results indicate that the use of good-quality photo/video displays for attacking can increase the possibility of successful attack of a face recognition system using MLBP image features, while the poor capturing condition can result in false rejection of real images as the presentation attack ones.

**Figure 16:**
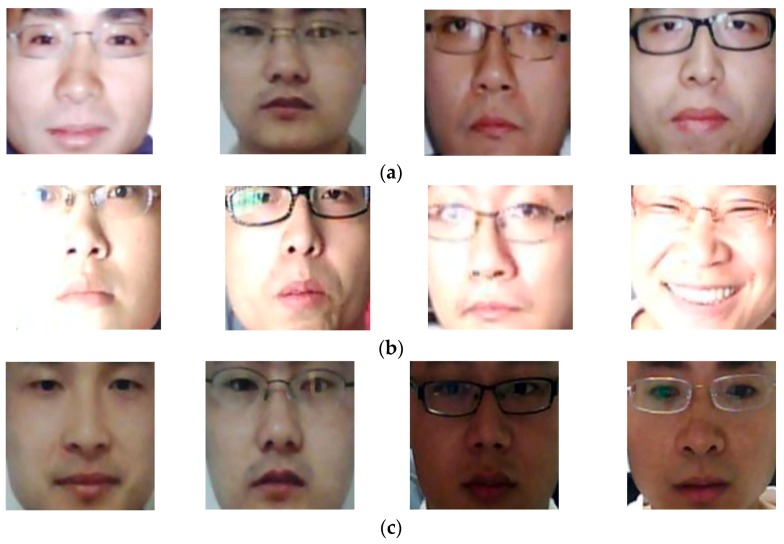
Examples of detection result images of the PAD method that uses only deep features on NUAA database: (**a**) “real to presentation attack” error cases, (**b**) “presentation to real” error cases, and (**c**) correct detection cases.

In Figure 16, we showed some examples of detection results of PAD method that only uses the deep features on NUAA database. Similar to Figure 15, we also showed the three examples of “presentation attack to real” classification error cases in Figure 16a, “real to presentation attack” classification error cases in Figure 16b, and the correct detection cases in Figure 16c. It is easily to observe from Figure 16b that the “real to presentation attack” classification error cases caused by the PAD method that only uses deep features contains large illumination variation. As shown in this figure, the non-uniform illumination occurred on the real images randomly on face regions. In addition, as shown in Figure 15 and Figure 16, the face images contain large texture variation caused by the background, glasses, and facial expression. Consequently, the face region contains very large variation compared to other biometric features such as finger-vein, finger-print, or iris. Although the CNN method has proven as a very powerful method for image classification and feature extraction, it still has its own limitations as explained in Section 2.4, especially the over-fitting problem, due to a huge amount of network’s parameters need to be trained. Therefore, the CNN method requires a huge amount of image data to train a model. As shown in Section 3.1, although we performed data augmentation on training database, the number of individuals in training database is still small (9 persons in NUAA and 20 persons in CASIA database).

As a result, the training data is small, and it can cause errors while training CNN models. In addition, as shown in Figure 16b,c, the PAD method can give correct detection results if the input face images do not contain extraordinary features such as facial expression and/or expensive illumination. By presenting expensive illumination on face, the texture features on face region can be disappeared and replaced by a plane or white texture. As a result, PAD method can produce incorrect detection results.

**Figure 17:**
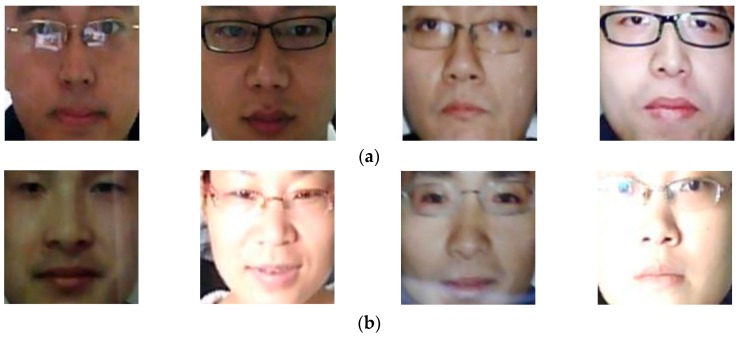
Examples of detection result images by our proposed PAD method on NUAA database: (**a**) images detected incorrectly by the PAD method that uses only MLBP features, but correctly detected by our proposed PAD method, and (**b**) images detected incorrectly by the PAD method that uses only deep features, but correctly detected by our proposed PAD method.

Figure 17 shows some examples of result images of our proposed PAD method. Figure 17a shows some result images that are incorrectly classified by the PAD method that uses only MLBP features, but correctly classified by our proposed method. Similarly, Figure 17b shows some result images that are incorrectly classified by the PAD method that uses only deep features, but correctly classified by our proposed method. As shown in this figure, although these images contain negative effects such as large variation of illumination or high quality of face region, they are correctly classified by our proposed method. This figure again shows the efficiency of our proposed PAD method over those that use only MLBP or only deep features.

As shown in Table 5 and Table 7, the performances of the PAD method that uses single feature extraction method vary according to the database used. In detail, the detection errors vary between the NUAA and CASIA databases with ACERs of 0.667% and 11.247% using handcrafted features on the NUAA database, and 10.566% and 2.174% using deep features on the CASIA database. These results show that the PAD method that only uses handcrafted image features or only deep image features has low reliability in application. However, the combination of these two features helps us in reducing the error rates in both databases as well as the difference between them (0.456% for NUAA database and 1.696% for CASIA). From this result, we can conclude that our proposed method has higher reliability than the PAD method that uses single feature extraction method.

## 5. Conclusions

In this paper, we have proposed a new method for detecting presentation attack images in a face recognition system to enhance its security level. Our proposed method is based on the use of hybrid features, which are a combination of handcrafted features and deep features, in order to collect richer information than that obtained using single feature extraction method. Our experiments indicated that the handcrafted image features are suitable for detecting presentation attack images with low image quality, while the deep image features are suitable for detecting presentation attack images with high quality. Thus, by combining the two types of image features, we can significantly enhance the detection accuracy compared to the use of a single method and other previous methods. In detail, in the case of using the NUAA database, which contains low-quality images and large illumination variation, the handcrafted features work better than deep features by yielding errors (ACERs) of 0.667% and 11.247% using MLBP and deep features, respectively. Using our proposed method, the error is significantly reduced to 0.456%. In the case of using the CASIA database, which is larger and contains higher-quality images as compared to the NUAA database, the deep features work better than handcrafted features. The detection errors (ACERs) generated using the CASIA database were 10.566%, 2.174%, and 1.696% using handcrafted features, deep features, and hybrid features, respectively. The experimental results indicated that our proposed method outperforms previous PAD methods for face recognition system using the same database (NUAA and CASIA databases).

## Figures and Tables

**Table 1:** Comparison of previous research on PAD problem for a face recognition system and our proposed method.

Category	Method	Strength	Weakness
**Non-training-based feature extraction methods**	- Using sparse low-rank bilinear discriminative model [14]; Gabor filtering, LBP features, and LPQ features [15,20]; Color texture information based on LBP method [17]; and DLTP features [21]. - Using image quality assessment [19]	- Easy to implement - Suitable for detecting low-quality presentation attack images	- Detection accuracy is fair - Detection accuracy may vary according to the image database
**Training-based feature extraction methods**	- Using CNN method with structure and parameter optimization [10] - Using handcrafted features and multiple pre-trained CNN models for feature extraction, and SVM method classification [34]. - Using multiple pre-trained CNN models for deep feature extraction; Combining deep and handcrafted image features using score-level fusion method [35]	- Learn the features for discriminating the real and presentation attack images automatically using a large number of training images [10] - Extract deep image features easily using pre-trained CNN models which were trained for other problems with handcrafted features [34] - Combines the strength of handcrafted and deep features for the detection problem; high detection accuracy [35]	- Requires time-consuming and powerful hardware for training a CNN model; Requires a large number of training images [10] - Requires multiple CNN and SVM models for extracting image features, and classification [34] - More complex than conventional methods; requires long processing time because of the use of multiple CNN and SVM models.
	- Using single CNN model; fine-tunes CNN model using a large number of images to train deep feature extractor; combines deep and handcrafted image features using score-level fusion and feature-level fusion methods. (**Proposed method**)	- More simple than previous deep learning-based methods by the use of single CNN model - Combines the strength of handcrafted and deep features for the detection problem - Archives better detection accuracy than single method (handcrafted or deep method)	- Complex and requires considerable processing power as compared to single handcrafted method or CNN method - Requires a large number of training images

**Table 2:** Description of the CNN architecture (based on VGG Net-19 network architecture) used in our study for PAD for a face recognition system.

Layer Name	Number of Filters	Filter Size	Stride Size	Padding Size	Dropout Value	Output Size
Input Layer	n/a	n/a	n/a	n/a	n/a	224 × 224 × 3
Convolution Layer (conv1_1)	64	3 × 3 × 3	1 × 1	1 × 1		224 × 224 × 64
ReLU (relu1_1)	n/a	n/a	n/a	n/a		224 × 224 × 64
Convolution Layer (conv1_2)	64	3 × 3 × 64	1 × 1	1 × 1		224 × 224 × 64
ReLU (relu1_2)	n/a	n/a	n/a	n/a		224 × 224 × 64
MAX Pooling Layer (pool1)	1	2 × 2	2 × 2	0		112 × 112 × 64
Convolution Layer (conv2-1)	128	3 × 3 × 64	1 × 1	1 × 1		112 × 112 × 128
ReLU (relu2_1)	n/a	n/a	n/a	n/a		112 × 112 × 128
Convolution Layer (conv2_2)	128	3 × 3 × 128	1 × 1	1 × 1		112 × 112 × 128
ReLU (relu2_2)	n/a	n/a	n/a	n/a		112 × 112 × 128
MAX Pooling Layer (pool2)	1	2 × 2	2 × 2	0		56 × 56 × 128
Convolution Layer (conv3_1)	256	3 × 3 × 128	1 × 1	1 × 1		56 × 56 × 256
ReLU (relu3_1)	n/a	n/a	n/a	n/a		56 × 56 × 256
Convolution Layer (conv3_2)	256	3 × 3 × 256	1 × 1	1 × 1		56 × 56 × 256
ReLU (relu3_2)	n/a	n/a	n/a	n/a		56 × 56 × 256
Convolution Layer (conv3_3)	256	3 × 3 × 256	1 × 1	1 × 1		56 × 56 × 256
ReLU (relu3_3)	n/a	n/a	n/a	n/a		56 × 56 × 256
Convolution Layer (conv3_4)	256	3 × 3 × 256	1 × 1	1 × 1		56 × 56 × 256
ReLU (relu3_4)	n/a	n/a	n/a	n/a		56 × 56 × 256
MAX Pooling Layer (pool3)	1	2 × 2	2 × 2	0		28 × 28 × 256
Convolution Layer (conv4_1)	512	3 × 3 × 256	1 × 1	1 × 1		28 × 28 × 512
ReLU (relu4_1)	n/a	n/a	n/a	n/a		28 × 28 × 512
Convolution Layer (conv4_2)	512	3 × 3 × 512	1 × 1	1 × 1		28 × 28 × 512
ReLU (relu4_2)	n/a	n/a	n/a	n/a		28 × 28 × 512
Convolution Layer (conv4_3)	512	3 × 3 × 512	1 × 1	1 × 1		28 × 28 × 512
ReLU (relu4_3)	n/a	n/a	n/a	n/a		28 × 28 × 512
Convolution Layer (conv4_4)	512	3 × 3 × 512	1 × 1	1 × 1		28 × 28 × 512
ReLU (relu4_4)	n/a	n/a	n/a	n/a		28 × 28 × 512
MAX Pooling Layer (pool4)	1	2 × 2	2 × 2	0		14 × 14 × 512
Convolution Layer (conv5_1)	512	3 × 3 × 512	1 × 1	1 × 1		14 × 14 × 512
ReLU (relu5_1)	n/a	n/a	n/a	n/a		14 × 14 × 512
Convolution Layer (conv5_2)	512	3 × 3 × 512	1 × 1	1 × 1		14 × 14 × 512
ReLU (relu5_2)	n/a	n/a	n/a	n/a		14 × 14 × 512
Convolution Layer (conv5_3)	512	3 × 3 × 512	1 × 1	1 × 1		14 × 14 × 512
ReLU (relu5_3)	n/a	n/a	n/a	n/a		14 × 14 × 512
Convolution Layer (conv5_4)	512	3 × 3 × 512	1 × 1	1 × 1		14 × 14 × 512
ReLU (relu5_4)	n/a	n/a	n/a	n/a		14 × 14 × 512
MAX Pooling Layer (pool5)	1	2 × 2	2 × 2	0		7 × 7 × 512
Fully Connected Layer (fc6)	n/a	n/a	n/a	n/a		4096
ReLU (relu6)						4096
Dropout Layer (drop6)					0.50	4096
Fully Connected Layer (fc7)					n/a	4096
ReLU (relu7)						4096
Dropout Layer (drop7)					0.50	4096
Output Layer (fc8)					n/a	2
Softmax Layer (prob.)						2
Classification Layer (output)						2

**Table 3:** Description of the NUAA database and its augmented databases used in our study.

Database	Training Database		Testing Database		Total (Training/Testing)
	Real Access	Presentation Attack	Real Access	Presentation Attack	
Original NUAA Database	1743	1748	3362	5761	(3491/9123)
Augmented NUAA Database	43,575 (1743 × 25)	43,700 (1748 × 25)	3362	5761	(87,275/9123)

**Table 4:** Description of CASIA database and its augmented databases used in our study.

Database	Training Database		Testing Database		Total (Training/Testing)
	Real Access	Presentation Attack	Real Access	Presentation Attack	
Original CASIA Database	10,914	34,138	15,910	49,712	45,052/65,622
Augmented CASIA Database	21,828 (10,914 × 2)	68,276 (34,138 × 2)	15,910	49,712	90,104/65,622

**Table 5:** Detection accuracy (in terms of APCER, BPCER, and ACER) of the PAD method that uses only handcrafted features on the NUAA and CASIA databases (unit: %).

Database	SVM Kernel	Without PCA			With PCA			
		APCER	BPCER	ACER	APCER	BPCER	ACER	No. PC
NUAA Database	Linear Kernel	2.604	2.380	2.492	0.972	3.183	2.077	90
	RBF Kernel	1.597	31.440	16.518	0.712	1.220	0.966	310
	Polynomial Kernel	1.840	31.916	16.878	0.590	0.744	0.667	310
CASIA Database	Linear Kernel	11.904	13.53	12.717	12.294	11.717	12.006	590
	RBF Kernel	8.8309	12.176	10.504	8.862	12.450	10.656	550
	Polynomial Kernel	8.5355	12.838	10.687	9.610	11.522	10.566	530

**Table 6:** Parameters of SGD method for training CNN model in our experiments using transfer learning technique.

Mini-Batch Size	Initial Learning Rate	Learning Rate Drop Factor	Learning Rate Drop Period (Epochs)	Training Size (Epochs)	Momentum
32	0.001	0.1	2	6	0.9

**Table 7:** Detection accuracy (in terms of APCER, BPCER, and ACER) of the PAD method that uses only deep features on NUAA and CASIA databases (unit: %).

Database	SVM Kernel	Without PCA			With PCA			
		APCER	BPCER	ACER	APCER	BPCER	ACER	No. PC
NUAA Database	Linear Kernel	7.915	22.219	15.067	6.909	15.586	11.247	40
	RBF Kernel	9.130	20.583	14.857	5.034	20.226	12.630	20
	Polynomial Kernel	8.367	20.851	14.609	7.412	23.587	15.500	30
CASIA Database	Linear Kernel	3.143	1.645	2.398	2.652	1.770	2.211	80
	RBF Kernel	3.067	1.756	2.412	3.369	2.183	2.776	70
	Polynomial Kernel	3.218	1.881	2.550	3.344	2.004	2.174	240

**Table 8:** Detection accuracy (in terms of APCER, BPCER, and ACER) of our proposed PAD method on NUAA and CASIA databases using feature-level fusion method (unit: %).

Database	SVM Kernel	Without PCA			With PCA			
		APCER	BPCER	ACER	APCER	BPCER	ACER	No. PC
NUAA Database	Linear Kernel	6.145	14.010	10.077	1.788	1.755	1.771	150
	RBF Kernel	3.888	25.937	14.913	0.174	42.683	21.428	10
	Polynomial Kernel	6.665	15.021	10.843	0.555	0.357	0.456	460
CASIA Database	Linear Kernel	2.784	1.629	2.207	2.037	1.356	1.696	120
	RBF Kernel	2.766	1.613	2.189	1.879	1.710	1.795	110
	Polynomial Kernel	2.753	1.647	2.200	2.037	1.513	1.775	210

**Table 9:** Detection accuracy (in terms of APCER, BPCER, and ACER) of our proposed PAD method on NUAA and CASIA databases using score-level fusion method (unit: %).

Database	SVM Kernel	Without PCA			With PCA		
		APCER	BPCER	ACER	APCER	BPCER	ACER
NUAA Database	Linear Kernel	2.500	2.282	2.391	2.202	1.951	2.077
	RBF Kernel	6.131	22.631	14.381	0.565	1.185	0.875
	Polynomial Kernel	5.417	20.784	13.100	0.476	0.784	0.630
CASIA Database	Linear Kernel	3.124	1.332	2.228	3.180	1.181	2.181
	RBF Kernel	3.080	1.487	2.283	2.665	1.973	2.319
	Polynomial Kernel	3.344	1.229	2.286	2.407	1.177	1.792

**Table 10:** Comparison of detection error (ACER) of our proposed PAD method with various previous methods using NUAA database (unit: %).

Baseline Method [14]	Gabor [15]	LPQ [15]	DLTP [21]	LBP [15]	LBP + Fisher Score + SVM [20]	Score-Fusion of Deep and Handcrafted Features [34,35]	Proposed Method
9.500	9.500	4.600	3.500	2.900	1.000	0.630	0.456

**Table 11:** Comparison of detection errors (ACERs) of our proposed PAD method with various previous methods using CASIA database (unit: %).

Baseline Method [16]	Combination of LBP, Fisher Score, and SVM [20]	Color Texture based on LBP Method [17]	DLTP [21]	Patch-Based Classification Method [23]	Score-Fusion of Deep and Handcrafted Features [34,35]	Proposed Method
17.000	13.100	6.200	5.400	5.07	1.792	1.696

**Table 12:** The processing time of our proposed method (unit: ms).

Face Detection and Normalization	Feature Extraction by MLBP Method	Feature Extraction by CNN Method	Feature Selection by PCA Method	Classification by SVM Method	Total Processing Time
16.956	12.915	19.519	0.792	0.320	50.502

**Table 13:** Description of the three sub-databases of CASIA database according to quality of face region and their augmented database used in our study.

Database According to Quality of Face Regions		Training Database		Testing Database		Total (Training/Testing)
		Real Access	Presentation Access	Real Access	Presentation Access	
Low Quality Database	Original Database	3140	11,019	5298	16,174	14,159/21,472
	Augmented Database	12,560	44,076	5298	16,174	56,636/21,472
Normal Quality Database	Original Database	3197	11,276	4830	16,157	14,473/20,987
	Augmented Database	12,788	45,104	4830	16,157	57,892/20,987
High Quality Database	Original Database	4577	11,843	5782	17,381	16,420/23,163
	Augmented Database	18,308	47,372	5782	17,381	65,680/23,163

**Table 14:** Detection accuracies of our proposed PAD method on sub-databases of CASIA database according to the image quality (unit: %).

Database	SVM Kernel	APCER	BPCER	ACER
Low Quality Database	Linear Kernel	3.718	0.742	2.230
	RBF Kernel	3.114	0.643	1.879
	Polynomial Kernel	1.906	1.762	1.834
Normal Quality Database	Linear Kernel	4.472	4.209	4.340
	RBF Kernel	2.836	5.063	3.950
	Polynomial Kernel	4.576	4.444	4.510
High Quality Database	Linear Kernel	0.882	3.958	2.420
	RBF Kernel	1.211	3.210	2.210
	Polynomial Kernel	0.796	4.102	2.449

**Table 15:** Description of the three sub-databases of CASIA database according to attacking methods (wrap photo, cut photo, and video display) and their augmented database used in our study.

Database According to Attack Method		Training Database		Testing Database		Total (Training/Testing)
		Real Access	Presentation Access	Real Access	Presentation Access	
Wrap Photo Database	Original Database	10,914	12,860	15,910	19,250	23,774/35,160
	Augmented Database	43,656	51,440	15,910	19,250	95,096/35,160
Cut Photo Database	Original Database	10,914	9,499	15,910	14,801	20,413/30,711
	Augmented Database	43,656	37,996	15,910	14,801	81,652/30,711
Video Display Database	Original Database	10,914	11,779	15,910	15,661	22,693/31,571
	Augmented Database	43,656	47,116	15,910	15,661	90,772/31,571

**Table 16:** Detection accuracies of our proposed PAD method on sub-databases of CASIA database according to the attacking methods (unit: %).

Database	SVM Kernel	APCER	BPCER	ACER
Wrap Photo Database	Linear Kernel	2.244	2.888	2.566
	RBF Kernel	2.206	1.901	2.054
	Polynomial Kernel	2.520	2.390	2.455
Cut Photo Database	Linear Kernel	1.113	0.203	0.658
	RBF Kernel	0.905	0.263	0.584
	Polynomial Kernel	0.949	0.142	0.545
Video Access Database	Linear Kernel	5.506	4.163	4.835
	RBF Kernel	5.0409	5.491	5.266
	Polynomial Kernel	4.6574	5.159	4.908

**Table 17:** Comparison of detection errors (ACERs) of our proposed PAD method with previous methods using CASIA sub-databases according to image quality and attacking methods (unit: %).

Detection Method	Low Quality Database	Normal Quality Database	High Quality Database	Wrap Photo Database	Cut Photo Database	Video Access Database
Baseline Method [16]	13.0	13.0	26.0	16.0	6.0	24.0
LBP-TOP [22]	10.0	12.0	13.0	6.0	12.0	10.0
IQA [23]	31.7	22.2	5.6	26.1	18.3	34.4
Combination of LBP, Fisher Score, and SVM [20]	7.2	8.8	14.4	12.0	10.0	14.7
Patch-based Classification Method [23]	5.26	6.00	5.30	5.78	5.49	5.02
Color Texture based on LBP method [17]	7.8	10.1	6.4	7.5	5.4	8.4
**Proposed Method**	1.834	3.950	2.210	2.054	0.545	4.835
